# Single-arm trials for domestic oncology drug approvals in China

**DOI:** 10.20892/j.issn.2095-3941.2023.0360

**Published:** 2023-11-27

**Authors:** Hong Zhang, Sen Liu, Chenghao Ge, Xiaozhen Liu, Yang Liu, Chen Yin, Yi Li, Jing An, Zhongtian Yan, Xiaoyuan Chen

**Affiliations:** 1 Center for Drug Evaluation, National Medical Products Administration, Beijing 100076, China; 2 Tsinghua Clinical Research Institute (TCRI), School of Medicine, Tsinghua University, Beijing 100084, China; 3 Office of Clinical Trial Institute, Beijing Tsinghua Changgung Hospital, Beijing 102218, China

The urgent need for effective cancer treatments, particularly for advanced and relapsed cases in which standard therapies are inadequate, has spurred the development of innovative therapeutic drugs^1,2^. Among the strategies to expedite drug development, the use of single-arm trials (SATs) is emerging as a promising avenue with substantial potential to shorten drug approval timelines and accelerate market entry. These trials, distinguished by their omission of parallel control groups and their incorporation of open designs devoid of randomization or blinding, have garnered favorable comparisons to conventional randomized controlled trials, because of their accelerated development timelines. In contrast, many patients with end-line tumors or rare tumors do not have standard treatments available; designing a control group may be impractical; and for ethical reasons, SATs may be more appropriate. Regulatory agencies are taking an active role in providing guidance, as exemplified by China, the United States, and the European Union, which have established conditional approval frameworks aligned with pressing healthcare needs^3–5^. The FDA granted conditional approvals based on SATs for 116 oncology indications from 2002 to 2021 in United States^6^, and China granted 53 such approvals between 2015 and 2022^7^. This editorial discusses considerations regarding the applicability of SATs in innovative domestic oncology drugs in China, from the perspective of regulatory considerations, and highlights the future challenges and limitations of applying SATs to support drug approval.

## SAT mapping

**Figure 1:**
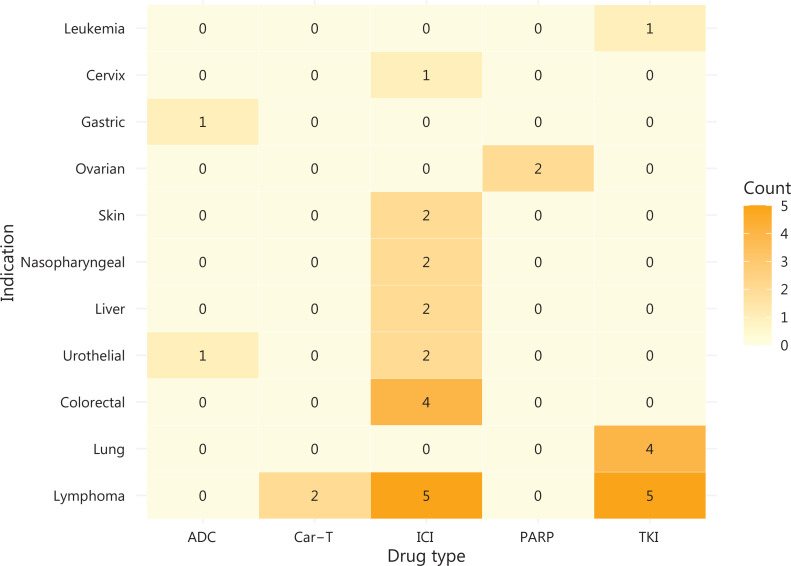
China’s innovative domestic oncology drugs approved on the basis of SATs, by drug type/indication, 2018–2022. ADC, antibody-drug conjugate; CAR-T, chimeric antigen receptor T-cell immunotherapy; ICI, immune checkpoint inhibitor; TKI, tyrosine kinase inhibitor.

We conducted a retrospective analysis of all new Chinese domestic oncology drugs that received regulatory approval on the basis of SATs between 2018 and 2022. As of December 31, 2022, the National Medical Products Administration (NMPA) had granted approval for 81 new oncology indications for domestic drugs. Of those, 34 (42%) were established through SATs (**Figure 1**): 21 (62%) of which pertained to new molecular entities (NMEs) or original biologics, and 13 (38%) of which pertained to supplementary indications. Notably, 97% of SAT approvals (33 of 34) used the objective response rate (ORR) or complete response (CR) as the primary endpoint (**Supplementary Table S1**). Major cytogenetic response (McyR) and major hematologic response (MaHR) were achieved for chronic phase and accelerated phase chronic myeloid leukemia (CML). Approvals had varying disease sites, and treatments for lymphomas and lung tumors were those most frequently approved. Lymphomas accounted for nearly one-third (32%) of approvals, and were closely followed by lung cancers (12%). The dawn of pan-tumor approvals arrived in 2020 with envafolimab, an anti-programmed cell death protein 1/programmed death-ligand 1 (PD1/PDL1) antibody, which was followed by 3 approvals for microsatellite instability high (MSI-H)/deficient mismatch repair (dMMR) solid tumors. Immune checkpoint inhibitors, particularly PD1/PDL1, constituted the largest category (53%) and included the world’s first approval for a PD1/cytotoxic T-lymphocyte-associated protein 4 (CTLA-4) dual antibody (cadonilimab). Tyrosine kinase inhibitors (TKIs) played a critical role in the treatment of lung and lymphatic system cancers, and included Bruton’s tyrosine kinase (BTK), anaplastic lymphoma kinase (ALK), epidermal growth factor receptor (EGFR), BCR-ABL1, and C-met. The approval landscape also included 2 antibody-drug conjugates (ADCs), 2 poly ADP ribose polymerase (PARP) inhibitors, and 2 chimeric antigen receptor T-cell (CAR-T) therapies.

## Overview of historical comparators and statistical analysis

SATs play a critical role in expediting drug development and providing early treatment accessibility. However, the use of SATs requires a careful assessment of the delicate balance between therapeutic benefits and potential risks. The limitations of SATs introduce an element of uncertainty that requires subsequent confirmatory studies. To address this uncertainty, mitigate effects on the benefit-risk assessment, and ensure the responsible use of SATs, Center for Drug Evaluation (CDE) of NMPA (China) issued the “Technical Guidelines on the Applicability of Single Arm Clinical Trials for Use in Support of Oncology Drug Marketing Applications” in March of 2023^8^. This guidance rigorously defines the conditions under which SATs can be applied, outlining 6 key application scenarios in which their use may be appropriate (**Supplementary Table S1**).

Two of these scenarios relate to unmet medical needs, addressing cases in which no available therapy exists within the study population or for rare cancers. In our study, “no available therapy” was defined as the absence of approved treatment options or standard therapies at the time of submission of a new drug application (NDA). For indications with more than 2 approved treatments, drugs submitted before the first drug approval for the same indication are considered contemporaneous and are subject to the same assessment criteria. However, if no approved treatment has undergone confirmatory studies, the treatment is categorized as lacking available therapeutic options. Of the total 19 indications we analyzed, 10 indications, representing 20 drugs, had no available therapy at the time of NDA submission (**Supplementary Table S2**). Four drugs (3 for urothelial cancer and 1 for gastric cancer) had existing treatments that had limited efficacy, as seen in cases such as second-line treatment for advanced urothelial carcinoma or third-line treatment for advanced gastric cancer, in which standard chemotherapy has limited efficacy. In addition, 10 drugs were submitted when imported drugs had already received marketing approval, but domestic alternatives were not yet available, thus highlighting the urgent need for domestically available alternatives. In cases of rare cancer, most proposed indications (74%, 25 of 34) are for rare cancers, and some (12%, 4 of 34) are for targeted populations enriched with specific biomarkers [such as EGFR T790M or human epidermal growth factor receptor 2 (HER2)], which inherently have a small patient population. Only a minority (15%, 5 of 34) of the proposed indications were for non-rare cancers.

The remaining 4 scenarios are more closely associated with clear clinical benefit evidence, encompassing well-defined therapeutic mechanisms, outstanding efficacy, clear external control data, and controllable safety risk. Outstanding efficacy is particularly important with respect to historical controls with robust evidence from meta-analyses, systematic reviews, real-world data, and other sources of evidence-based medicine.

**Figure 2:**
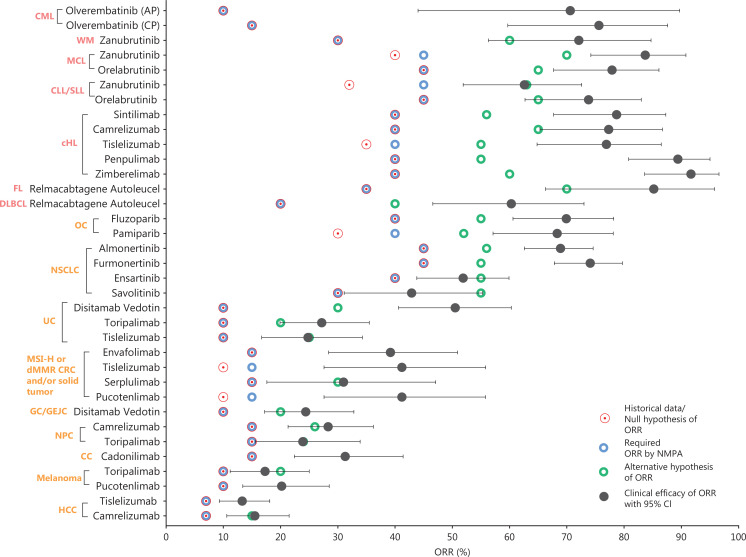
Overview of statistical analysis and clinical efficacy of ORR (or CR) for all domestic cancer drugs newly approved on the basis of SATs, 2018–2022. The L described in the indication represents the line of treatment. MSI-H, microsatellite instability high; dMMR, deficient mismatch repair; CRC, colorectal cancer; WM, Waldenstrom macroglobulinemia; ALK, anaplastic lymphoma kinase; NSCLC, non-small cell lung carcinoma; EGFR, epidermal growth factor receptor; DLBCL, diffuse large B-cell lymphoma; CLL, chronic lymphocytic leukemia; SLL, small lymphocytic leukemia; MCL, mantle cell lymphoma; HCC, hepatocellular carcinoma; PTCL, peripheral T-cell lymphoma; cHL, classical Hodgkin’s lymphoma; OC, ovarian cancer; NPC, nasopharyngeal carcinoma; UC, urothelium carcinoma; HER2, human epidermal growth factor receptor 2; GC/GEJC, gastric and gastroesophageal junction cancers; BC, breast cancer; CML, chronic granulocytic leukemia. The null hypothesis of ORR (or CR) was established as the minimum clinically meaningful efficacy, which was derived from the published pivotal trial data for the drug. Historical data derived from the regulatory review documents or published pivotal trial data can in some cases be used as the basis for the null hypothesis of ORR. The alternative hypothesis of ORR (or CR) was defined as the targeted efficacy, which was derived from the published pivotal trial data for the drug. The ORR required by NMPA was defined as the agreed lower limit of the efficacy point estimate of ORR (or CR) reported in the regulatory review documents.

Our study findings revealed that all approved single-arm studies used sample size calculations based on statistical assumptions, and specifically used historical control data as the reference for the null hypothesis regarding ORR values (**Figure 2**). Sample assumptions varied across tumors. The median sample size in approved single-arm studies was 102 (minimum: 28, maximum: 249). The sample size of most rare cancers were below 100. Most analyzed drugs (82%, 28 of 34) had preset null hypothesis ORR values, in accordance with the required efficacy standard set by the NMPA, whereas a minority (18%, 6 of 34) set the null hypothesis values above than those required by the NMPA. Further analysis revealed that the lower limits of the 95% confidence intervals for the actual efficacy values of all drugs were consistently higher than their respective null hypothesis values, and nearly half (41%, 14 of 34) of the drugs demonstrated lower limits of the 95% confidence intervals for actual efficacy values exceeding the statistically estimated alternative hypothesis regarding the ORR. Intriguingly, half (50%, 17 of 34) of the drugs had actual efficacy values surpassing twice the NMPA’s required efficacy. In general, the standard set by the NMPA would be the same, or within a similar range, for the same indication, but might vary by tumor type. Moreover, a general, gradual, and modest increase in the ORR over time was observed within the same indication (**Supplementary Figure S1**). Some individual cases showed lower ORR values than those of previously approved drugs, most of which were drugs developed contemporaneously with similar evaluation criteria. For instance, serplulimab, used as a second-line treatment for MSI-H or dMMR colorectal cancer (CRC) and/or solid tumors, exhibited slightly lower efficacy than envafolimab and tislelizumab, which were developed in parallel. However, the efficacy of serplulimab still surpassed the NMPA required efficacy by more than twofold. After categorization by origin and nature of the tumors, we observed that the ORR treatment efficacy for hematological malignancies exceeded that for solid tumors, in agreement with clinical experience. Thus, most domestic drugs approved through single-arm studies exhibit clinically significant improvements over previous treatment approaches.

Because most domestic drugs are concentrated in PD1/PDLI related immune checkpoint inhibitors or imported drugs of the same class that have been approved in China, the relevant drug treatment mechanism is relatively clear, and known safety-associated risks are also disclosed for imported drugs. The main adverse reaction characteristics of domestically approved PD1 drugs are similar to those of similar products or those in other indications. The safety profiles of “first-in-class” drugs in China, such as the C-met kinase inhibitor (savolitinib) and HER2-ADC (disitamab vedotin), align with those reported in international clinical studies. Similarly, in the case of global “first-in-class” drugs, no significant difference in adverse reactions has been observed for PD1/CTLA-4 dual antibody (cadonilimab), compared with PD1 and CTLA-4 monotherapies. All domestic drugs have a corresponding risk control plan at the time of NDA submission, coupled with an extensive regime of post-marketing safety surveillance; consequently, the overall safety risks of domestic oncology drugs are generally controllable.

## Confirmatory trials and converted approval

Drugs granted conditional approval on the basis of SATs in oncology often undergo subsequent confirmatory trials, which generally fall into 3 distinct categories: randomized controlled trials (RCTs), extended single-arm studies with larger sample sizes, and real-world studies. We found that only 1 drug, relmacabtagene autoleucel, which was recently approved as a third-line treatment for r/r FL, has not yet initiated its confirmatory trial, whereas all other drugs in our study have commenced confirmatory trials (**Supplementary Table S1**). A substantial majority (85%, 28 of 33) of the approved drugs had RCTs selected as the chosen approach for the confirmatory trials. Within this subset, less than half (39%, 11 of 28) used active controls, several (32%, 9 of 28) involved comparison with standard care, and the remaining were placebo controlled; almost all studies (96%, 27 of 28) used a primary clinical endpoint of progression-free survival (PFS), whereas only 1 study [of disitamab vedotin used as a third-line treatment for HER2(+) GC/GEJC] used OS as the primary endpoint. In contrast, for pan-tumor studies, indications such as MSI-H or dMMR solid tumors, or for studies including rare cancer types, such as MET Ex14 skipping NSCLC or r/r DLBCL, the design of confirmatory studies tends to favor extended single-arm studies essentially constituting a continuation of the original study. Notably, we identified no examples of use of real-world evidence as the basis for confirmatory trials among approved domestic drugs.

In addition, a substantial proportion (71%, 24 of 34) of participants in these confirmatory trials moved into the front-line treatment population. The remaining cases involved continuation of pivotal studies for specific indications, such as MSI-H or dMMR solid tumors, as well as the maintenance of r/r cHL, r/r DLBCL and CML, or HER2(+) GC/GEJC regimens in line with previous regimens. Notably, nearly half (48%, 14 of 29) of the approved drugs had carefully planned confirmatory trials in the development phase, which began before the formal NDA submission.

As of July 2023, 9 domestic oncology drugs have converted full approvals, defined as successful completion of confirmatory studies and submitting NDAs or supplemental applications. Additionally, tislelizumab, intended as a first-line treatment for hepatocellular carcinoma (HCC), is currently undergoing review, and its NDA was submitted in December 2022 (**Supplementary Table S3**). All 10 of these drugs were initially granted conditional approval for populations with no available therapy or imported alternatives, followed by conducting confirmatory studies in front-line populations, and ultimately had fully approved conversions and new approvals for their front-line indications. Notably, the initiation of the confirmatory trials of these drugs was conducted well, and occurred before the NDA submission for the previous indication. The time for full approval conversion ranged from as short as 2 months (for camrelizumab, used as a first-line treatment for NPC) to no longer than 4.6 years (for zanubrutinib, used as a first-line treatment for r/r CLL/SLL).

## Regulatory considerations in SATs for drug approval

Domestic oncology drugs supported by SATs for approval in China have all been granted conditional approval in the past 5 years. Considering the timeframe for completing post-marketing confirmatory trials within 5 years of being granted conditional approval, we anticipate that a wave of drugs might be submitted for post-approval supplementary applications in the next 2 years. Our analysis suggested that several companies might be at risk of delays in filing these supplementary applications. According to the FDA’s experience, approximately 50% of drugs completed confirmatory clinical trials, and 12% of indications were eventually withdrawn. The median time for conversion to full approval was 3.1 years, and the timing of completion of confirmatory trials was influenced by whether a confirmatory trial was initiated at the time of accelerated approval. Consequently, the FDA recommends early discussions regarding the design and initiation of confirmatory trials, preferably initiated before the time of application for accelerated approval or the completion of most patient enrollment^3^. The FDA may also withdraw conditional approval because of failure or delay of the completion of a confirmatory trial. However, in China, procedures for conditional drug approval were recently released in August 2023 and are currently in the phase of solicitation of opinions^9^. This guidance clarified the completion timeline for confirmatory studies, established comprehensive evaluation criteria, and outlined the process for withdrawal or conversion to full approval. The overarching concept aligns with that of the FDA, which is to withdraw drugs that do not meet the specified conditions. In China, certain criteria may be more stringent; notably, only a single drug with the same mechanism is permitted to receive conditional approved for a specific indication.

## Future challenges

Effective post-marketing risk management is particularly important for drugs approved on the basis of SATs. Because of limitations in trial design, and constraints on the premarket sample size and observation period, fully identifying the safety risks of a product might not be possible. These risks include a low incidence of adverse reactions, potential long-term safety risks, or a risk of drug ineffectiveness. Consequently, certain drugs may require extended safety monitoring after market approval. For instance, relmacabtagene autoleucel, a cell gene therapy, requires a 15-year follow-up period to observe potential long-term oncogenic risks.

The assessment of therapeutic effectiveness in SAT often relies on comparisons with historical control data. However, achieving a consistent baseline between a historical control group and SAT participants can be challenging. Factors such as demographic variables, comorbidities, disease characteristics (such as severity, symptoms, and progression), initiation of treatment follow-up, concurrent therapies, and collected clinical observations can introduce biases into the evaluation of therapeutic efficacy. To address these issues, the FDA encourages the inclusion of real-world studies as a valid external control, and supplementary evidence from SATs to substantiate the safety and efficacy of a drug^10^. Nonetheless, in designing external control trials, careful consideration must be paid to factors that ensure the comparability of data with the SAT, including the timing and frequency of data collection, therapeutic regimen, healthcare practices, and criteria for assessing patient outcomes.

The acceptance of the inherent risk of uncertainty associated with SAT changes as post-marketing evidence accumulates, and the criteria for the use of SATs should be adjusted periodically in light of emerging evidence, as exemplified in April 2021, when the FDA’s Oncology Drugs Advisory Committee reevaluated 6 PI3K inhibitors that had initially received accelerated approval through single-arm pathways. Some of these drugs were withdrawn from the market because of safety concerns, and subsequent PI3K inhibitors were required to undergo RCT studies for approval^11^. Correspondingly, recent guidelines for SATs issued by the China’s CDE emphasize that the applicable conditions for supporting anti-tumor drug approval in SATs should be “patient-centered” and “clinically value-oriented”^8,12,13^. Moreover, the acceptance of uncertainty risks must adapt to the evolving landscape of drug development and clinical practice, while ensuring that the benefits consistently exceed the uncertainties, to ultimately optimize the benefit-risk ratio.
