# Supplementary material for: Single-arm trials for domestic oncology drug approvals in China
Source: Cancer Biol Med. 2023 Nov 27;20(11):799–805. doi: 10.20892/j.issn.2095-3941.2023.0360 (PMC10690876; doi:10.20892/j.issn.2095-3941.2023.0360)
Supplement: Supplementary file 1 [file cbm-20-799-s001.pdf]

Supplementary materials

Methods

A retrospective analysis was conducted on all new Chinese domestic oncology drugs that received regulatory approval on the basis of SATs between January 2018 and December 2022. NDAs for oncology therapeutics (new molecular entities or novel biologics) developed primarily by pharmaceutical companies within China were included, excluding non-therapeutic (e.g., diagnostics), cancer care, licensed-in (developed overseas but licensed to Chinese companies), and generic drugs. SATs described in this article were applicable to only single agents rather than combination therapies for refractory or rare tumors. Data on the clinical efficacy and registration information were obtained primarily from publicly available regulatory review documents. The collected data were analyzed with descriptive statistics, and are presented in tables and figures to illustrate the overall landscape of China's domestic oncology drug regulation.

Supplementary results

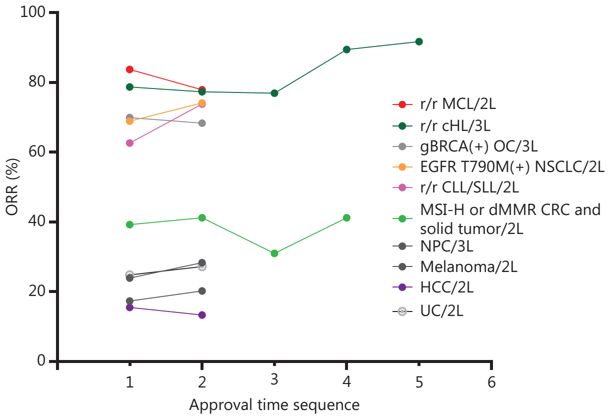

Figure S1 Objective response rate (ORR) of the domestic-matched Chinese domestic oncology drugs by approval time sequence. Approved drugs with different indications are distinguished by color coding. The L described in the indication represents the line of treatment. The r/r described in the indication represents refractory and relapse. MSI-H, microsatellite instability high; dMMR, deficient mismatch repair; CRC, colorectal cancer; WM, Waldenstrom macroglobulinemia; ALK, anaplastic lymphoma kinase; NSCLC, non-small cell lung carcinoma; EGFR, epidermal growth factor receptor; CLL, chronic lymphocytic leukemia; SLL, small lymphocytic leukemia; MCL, mantle cell lymphoma; HCC, hepatocellular carcinoma; cHL, classical Hodgkin's lymphoma; OC, ovarian cancer; NPC, nasopharyngeal carcinoma; UC, urothelium carcinoma.

Table S1 Considerations regarding the applicability of single-arm clinical trials to supporting oncology drug approval

No.	Application scenarios	Description
1	No available therapy	SATs will be considered when an urgent need exists to treat patients with tumors for which no treatment options are available (e.g., advanced refractory recurrent tumors, tumors for which no standard treatment exists, or tumors refractory to standard treatment), However, each participant must be ensured to meet the criteria of having no available therapy.
2	Clear mechanism of drug treatment	SATs can be applied if the tumor pathogenesis is clear, and the mechanism of drug action is also clear and consistent with the etiology.
3	Clear external control data	Because of the absence of a parallel control group in SATs, sufficient evidence from evidence-based medicine as historical controls must be used. The data sources can be an individual RCT, systematic review studies, meta-analysis data, or even a real-world study.
4	Outstanding efficacy	For drugs with outstanding efficacy, tolerating the uncertainty introduced by SATs is acceptable. In SATs, not only the ORR but also data such as DOR and PFS, which may be associated with survival benefits, should be assessed.
5	Controllable safety risk	Early safety data should reflect the safety profile of the investigational drug. If early data show characteristics such as a high mortality rate, drug discontinuation, dose adjustments, or interruptions due to adverse events, or if patients become intolerant because of long-term adverse events, and the incidence of severe adverse events, or grade 3 or 4 adverse events is significantly higher than that in similar treatment populations, cautious consideration the feasibility of conducting a SAT is advisable.
6	Rare cancer	In the case of rare tumors for which conducting an RCT is challenging, SATs may be considered a pivotal study in support of approval. However, the study must be based on evidence of disease characteristics, pathogenesis, and the mechanism of drug action. Notably, SATs may not be applicable to a small number of patients.

Table S2 Characteristics of newly approved domestic anti-cancer drugs with marketing application supported by single-arm studies

Group	Indication	Biological pathway	Active ingredients	Available therapy	Rare cancer$	NDA filing time	Approval time (indications)	Primary outcome	Sample size	Registration number of the pivotal trial	Efficacy (95% CI) in pivotal trial	Required efficacy by NMPA*	Initiation time of the confirmatory trial#	Registration number of confirmatory trial	Full approval conversion (Y/N)
1	MSI-H or dMMR CRC and/or solid tumor/2L	PD1-PDL1 inhibitors	Envafolimab	No	Rare	2020.12	2021.11	ORR	103	NCT03667170	39.2% (28.4%, 50.9%)	15%	2018.08&	NCT03667170	N
			Tislelizumab			2021.06	2022.03	ORR	80	NCT03736889	41.2% (27.6%, 55.8%)	15%	2018.09&	NCT03736889	N
			Serplulimab			2021.04	2022.03	ORR	108	NCT03941574	31.0% (17.6%, 47.1%)	15%	2019.07&	NCT03941574	N
			Pucotenlimab			2021.10	2022.07	ORR	100	NCT03704246	41.2% (27.6%, 55.8%)	15%	2022.11	NCT05652894	N
2	r/r WM/2L	BTK inhibitors	Zanubrutinib	Imported available	Rare	2020.09	2021.06	ORR	44	NCT03332173	72.1% (56.3%, 84.7%)	30%	2017.01	NCT03053440	Y
3	ALK(+) NSCLC/2L	ALK-TKI inhibitors	Ensartinib	Imported available	Rare	2018.12	2020.11	ORR	160	NCT03215693	51.9% (43.8%, 59.9%)	40%	2016.06	NCT02767804	Y
4	EGFR T790M(+) NSCLC/2L	EGFR inhibitors	Almonertinib	Imported available	No	2019.04	2020.03	ORR	244	NCT02981108	68.9% (62.6%, 74.6%)	45%	2019.02	NCT03849768	Y
			Furmonertinib			2019.11	2021.03	ORR	220	NCT03452592	74.1% (67.8%, 79.7%)	45%	2019.05	NCT03787992	Y
5	Melanoma/2L	PD1-PDL1 inhibitors	Toripalimab	No	Rare	2018.03	2018.12	ORR	128	NCT03013101	17.3% (11.2%, 25.0%)	10%	2018.02	NCT03430297	N
			Pucotenlimab			2021.07	2022.09	ORR	119	NCT04749485	20.2% (13.4%, 28.5%)	10%	2022.11	NCT05647954	N
6	r/r DLBCL/3L	CD19 inhibitors	Relmacabtagene Autoleucel	No	Rare	2020.06	2021.09	ORR	59	NCT04089215	60.3% (46.6%, 73%)	20%	2022.03	CTR20220683	N
7	r/r CLL/SLL/2L	BTK inhibitors	Zanubrutinib	Imported available	Rare	2018.10	2020.06	ORR	91	NCT03206918	62.6% (51.9%, 72.6%)	45%	2017.11	NCT03336333	N
			Orelabrutinib			2019.11	2020.12	ORR	80	NCT03493217	73.8% (62.7%, 83.0%)	45%	2021.01	NCT04578613	N
8	r/r MCL/2L	BTK inhibitors	Zanubrutinib	Imported available	Rare	2018.08	2020.06	ORR	86	NCT03206970	83.7% (74.2%, 90.8%)	45%	2019.08	NCT04002297	N
			Orelabrutinib			2020.03	2020.12	ORR	86	NCT03494179	77.9% (67.7%, 86.1%)	45%	2021.12	NCT05051891	N
9	HCC/2L	PD1-PDL1 inhibitors	Camrelizumab	Imported available	No	2019.05	2020.03	ORR	220	NCT02989922	15.5% (10.6%, 21.5%)	7%	2019.06	NCT03764293	Y
			Tislelizumab			2020.06	2021.06	ORR	249	NCT03419897	13.3% (9.3%, 18.1%)	7%	2017.12	NCT03412773	N
10	r/r cHL/3L	PD1-PDL1 inhibitors	Sintilimab	No	Rare	2018.04	2018.12	ORR	96	NCT03114683	78.7% (67.7%, 87.3%)	40%	2019.10	NCT04044222	N
			Camrelizumab			2018.04	2019.05	ORR	75	NCT03155425	77.3% (65.3%, 86.7%)	40%	2020.07	NCT04342936	N
			Tislelizumab			2018.08	2019.12	ORR	70	NCT03209973	76.9% (64.8%, 86.5%)	40%	2020.09	NCT04486391	N
			Penpulimab			2020.05	2021.08	ORR	94	NCT03722147	89.4% (80.8%, 95.0%)	40%	2022.02	NCT05244642	N
			Zimberelimab			2020.02	2021.08	ORR	85	NCT03655483	91.7% (83.6%, 96.6%)	40%	2022.09	NCT05518318	N
11	gBRCA(+) OC/3L	PARP inhibitors	Fluzoparib	No	Rare	2019.10	2020.12	ORR	113	NCT03509636	69.9% (60.6%, 78.2%)	40%	2019.04	NCT03863860	Y
			Pamiparib			2020.07	2021.04	ORR	113	NCT03333915	68.3% (57.1%, 78.1%)	40%	2018.05	NCT03519230	N
12	NPC/3L	PD1-PDL1 inhibitors	Toripalimab	No	Rare	2020.05	2021.02	ORR	92	NCT02915432	23.9% (15.6%, 33.9%)	15%	2018.10	NCT03581786	Y
			Camrelizumab			2020.09	2021.04	ORR	156	NCT03558191	28.3% (21.3%, 36.2%)	15%	2018.11	NCT03707509	Y

Table S2 Continued

Group	Indication	Biological pathway	Active ingredients	Available therapy	Rare cancer[$]	NDA filing time	Approval time (indications)	Primary outcome	Sample size	Registration number of the pivotal trial	Efficacy (95% CI) in pivotal trial	Required efficacy by NMPA*	Initiation time of the confirmatory trial[#]	Registration number of confirmatory trial	Full approval conversion (Y/N)
13	PD-L1(+) UC/2L	PD1-PDL1 inhibitors	Tislelizumab	Yes, but limited	No	2019.06	2020.04	ORR	113	NCT04004221	24.8% (16.7%, 34.3%)	10%	2019.05	NCT03967977	N
	UC/2L		Toripalimab			2020.05	2021.04	ORR	151	NCT03113266	27.2% (19.9%, 35.5%)	10%	2020.09	NCT04568304	N
14	HER2(+) GC/GEJC/3L	HER2-ADC	Disitamab vedotin	Yes, but limited	No	2020.08	2021.06	ORR	127	NCT03556345	24.4% (17.2%, 32.8%)	10%	2020.09	CTR20202569	N
15	HER2(+) UC/2L	HER2-ADC	Disitamab vedotin	Yes, but limited	No	2021.07	2021.12	ORR	43<br>64	NCT03507166;<br>NCT03809013	50.5% (40.6%, 60.3%)	10%	2022.06	NCT05302284	N
16	MET Ex14 skipping NSCLC/2L	C-Met inhibitors	Savolitinib	No	Rare	2020.06	2021.06	ORR	76	NCT02897479	42.9% (31.1%, 55.3%)	30%	2021.08	NCT04923945	N
17	T315I(+) CML/2L	BCR-ABL1 inhibitors	Olverembatinib	No	Rare	2020.09	2021.11	McyR<br>MaHR	41<br>23	NCT03883087;<br>NCT03883100	75.6% (59.7%, 87.6%)<br>70.6% (44.0%, 89.7%)	15%<br>10%	2019.10	NCT04126681	N
18	CC/2L	PD1/CTLA-4 BsAb	Cadonilimab	No	No	2021.09	2022.06	ORR	111	NCT03852251	31.3% (22.4%, 41.4%)	15%	2021.08	NCT04982237	N
19	r/r FL/3L	CD19 inhibitors	Relmacabtagene Autoleucel	No	Rare	2022.03	2022.09	ORR	28	NCT04089215	85.2% (66.3%, 95.8%)	35%	NA	NA	N

The L described in the indication represents the line of treatment. The r/r described in the indication represents refractory and recurrent. ORR, objective response rate. OS, overall survival; PFS, progression-free-survival; McyR, major cytogenetic response; MaHR, major hematologic response; MSI-H, microsatellite instability high; dMMR, deficient mismatch repair; CRC, colorectal cancer; WM, Waldenstrom macroglobulinemia; ALK, anaplastic lymphoma kinase; NSCLC, non-small-cell lung carcinoma; EGFR, epidermal growth factor receptor; DLBCL, diffuse large B-cell lymphoma; CLL, chronic lymphocytic leukemia; SLL, small lymphocytic lymphoma; MCL, mantle cell lymphoma; HCC, hepatocellular carcinoma; PTCL, peripheral T-cell lymphoma; cHL, classical Hodgkin's lymphoma; OC, ovarian cancer; NPC, nasopharyngeal carcinoma; UC, urothelium carcinoma; HER2, human epidermal growth factor receptor 2; GC/GEJC, gastric and gastroesophageal junction cancers; BC, breast cancer; CML, chronic granulocytic leukemia; CC, cervical cancer; FL, follicular lymphoma. *Required efficacy by NMPA was defined as the agreed lower limits of the efficacy point estimate of ORR (or CR) reported in the regulatory review documents. [#]The initiation time of the confirmatory trials was collected as the actual start date of the trial on ClinicalTrials.gov. [&]The confirmatory trial was the continuation of the previous pivotal trial. [$]The definition of rare cancer was based on terms such as rare and uncommon, as described in the regulatory review report, as well as the relatively authoritative epidemiology-based definition of rare disease involving an incidence or prevalence rate less than 1 in 10,000 or a total number of cases less than 140,000.

Table S3 Characteristics of the confirmatory trial for domestic anti-cancer drugs undergoing full approval conversion

Group	Indication	Active ingredients	Study design	Type of blinding	Combination therapy	Sample size	Primary outcome	Efficacy	Study population	Approval time (new indications)	Front-line therapy (new indications) (Y/N)	Time for full approval conversion (year)
1	MSI-H or dMMR CRC and/or solid tumor/2L	Envafolimab	Single-arm	Open-label	No	200 (103)	ORR	/	MSI-H or dMMR CRC and/or solid tumor/2L	/	N	/
		Tislelizumab	Single-arm	Open-label	No	200 (80)	ORR	/		/	N	/
		Serplulimab	Single-arm	Open-label	No	108	ORR	/		/	N	/
		Pucotenlimab	Chemotherapy control	Open-label	No	190	PFS	/	MSI-H or dMMR CRC/1L	/	Y	/
2	r/r WM/2L	Zanubrutinib[1]	Active control (Ibrutinib)	Open-label	No	201	VGPR+CR	36.3% *vs* 25.3% (*P* = 0.07)	WM/1L	2023.05	Y	2.9
3	ALK(+) NSCLC/2L	Ensartinib	Active control (crizotinib)	Open-label	No	290	PFS	mPFS: 25.8 m *vs* 12.7 m HR (PFS): 0.56	ALK(+) NSCLC/1L	2022.03	Y	1.3
4	EGFR T790M(+) NSCLC/2L	Almonertinib	Active control (gefitinib)	Double-blind	No	429	PFS	mPFS: 19.3 m *vs* 9.9 m HR (PFS): 0.46	EGFR(+) Ex19del and/or Ex21L858R substitution NSCLC/1L	2021.12	Y	2.7
		Furmonertinib	Active control (gefitinib)	Double-blind	No	358	PFS	mPFS: 20.8 m *vs* 11.1 m HR (PFS): 0.44		2022.06	Y	2.6
5	Melanoma/2L	Toripalimab	Active control (dacarbazine)	Open-label	No	230	PFS	/	Melanoma/1L	/	Y	/
		Pucotenlimab	Active control (temozolomide)	Open-label	Transcatheter arterial chemoembolization	350	PFS	/	Stage IV melanoma/1L	/	Y	/
6	r/r DLBCL/3L	Relmacabtagene Autoleucel	Single-arm	Open-label	No	41	ORR	/	r/r DLBCL/3L	/	N	/
7	r/r CLL/SLL/2L	Zanubrutinib[2]	Active control (bendamustine+rituximab)	Open-label	No	740	PFS	HR (PFS): 0.42	CLL/SLL/1L	2023.05	Y	4.6
		Orelabrutinib	Active control (chlorambucil+rituximab)	Open-label	No	218	PFS	/		/	Y	/
8	r/r MCL/2L	Zanubrutinib	Active control (bendamustine+rituximab)	Open-label	Rituximab	500	PFS	/	MCL/1L	/	Y	/
		Orelabrutinib	R-CHOP add-on	Open-label	R-CHOP	356	PFS	/		/	Y	/
9	HCC/2L	Camrelizumab[3]	Active control (sorafenib)	Open-label	Apatinib	543	PFS	mPFS:5.6 m *vs* 3.7 m HR (PFS): 0.52	HCC/1L	2023.01	Y	2.8
		Tislelizumab	Active control (sorafenib)	Open-label	No	674	PFS	/		2022.12 (NDA)	Y	/
10	r/r cHL/3L	Sintilimab	Placebo control	Double-blind	ICE regimen	240	PFS	/	cHL/2L	/	Y	/
		Camrelizumab	Chemotherapy control	Open-label	No	56	PFS	/	r/r cHL/3L	/	N	/
		Tislelizumab	Chemotherapy control	Open-label	No	123	PFS	/	r/r cHL/3L	/	N	/
		Penpulimab	Chemotherapy control	Open-label	No	60	PFS	/	r/r cHL/3L	/	N	/
		Zimberelimab	Chemotherapy control	Open-label	No	60	PFS	/	r/r cHL/3L	/	N	/

Table S3 Continued

Group	Indication	Active ingredients	Study design	Type of blinding	Combination therapy	Sample size	Primary outcome	Efficacy	Study population	Approval time (new indications)	Front-line therapy (new indications) (Y/N)	Time for full approval conversion (year)
11	gBRCA(+) OC/3L	Fluzoparib	Placebo control	Double-blind	No	252	PFS	mPFS: 12.9 m *vs* 5.5 m HR (PFS): 0.25	rOC/2L maintenance	2021.06	Y	0.5
		Pamiparib	Placebo control	Double-blind	No	216	PFS	/		/	Y	/
12	NPC/3L	Toripalimab	Placebo control	Double-blind	Cisplatin+gemcitabine	289	PFS	mPFS: 11.7 m *vs* 8.0 m HR (PFS): 0.52	r/m NPC/1L	2021.11	Y	0.8
		Camrelizumab	Placebo control	Double-blind		231	PFS	mPFS: 9.7 m *vs* 6.9 m HR (PFS): 0.54		2021.06	Y	0.2
13	PD-L1(+) UC/2L	Tislelizumab	Placebo+chemotherapy control	Double-blind	Chemotherapy	420	PFS	/	UC/1L	/	Y	/
	UC/2L	Toripalimab	Placebo+chemotherapy control	Double-blind	Chemotherapy	364	PFS	/		/	Y	/
14	HER2(+) GC/GEJC/3L	Disitamab vedotin	Physician choice control	Open-label	No	351	OS	/	HER2(+) GC/GEJC/3L	/	N	/
15	HER2(+) UC/2L	Disitamab vedotin	Chemotherapy control	Open-label	Toripalimab	452	PFS	/	HER2(+) UC/1L	/	Y	/
16	MET Ex14 skipping NSCLC/2L	Savolitinib	Single-arm	Open-label	No	163	ORR	/	MET Ex14 skipping NSCLC/1L	/	Y	/
17	T315I(+) CML/2L	Olverembatinib	Best available therapy control	Open-label	No	144	PFS	/	CML-CP/2L	/	N	/
18	CC/2L	Cadonilimab	Placebo+chemotherapy ± bevacizumab control	Double-blind	Chemotherapy ± bevacizumab	440	PFS	/	CC/1L	/	Y	/

The L described in the indication represents the line of treatment. The r/r described in the indication represents refractory and recurrent. ORR, objective response rate; OS, overall survival; PFS, progression-free-survival; McyR, major cytogenetic response; MaHR, major hematologic response; MSI-H, microsatellite instability high; dMMR, deficient mismatch repair; CRC, colorectal cancer; WM, Waldenstrom macroglobulinemia; ALK, anaplastic lymphoma kinase; NSCLC, non-small-cell lung carcinoma; EGFR, epidermal growth factor receptor; DLBCL, diffuse large B-cell lymphoma; CLL, chronic lymphocytic leukemia; SLL, small lymphocytic lymphoma; MCL, mantle cell lymphoma; HCC, hepatocellular carcinoma; PTCL, peripheral T-cell lymphoma; cHL, classical Hodgkin's lymphoma; OC, ovarian cancer; NPC, nasopharyngeal carcinoma; UC, urothelium carcinoma; HER2, human epidermal growth factor receptor 2; GC/GEJC, gastric and gastroesophageal junction cancers; BC, breast cancer; CML, chronic granulocytic leukemia; CC, cervical cancer; FL, follicular lymphoma.

References

1. Dimopoulos MA, Opat S, D'Sa S, Jurczak W, Lee HP, Cull G, et al. Zanubrutinib versus ibrutinib in symptomatic waldenström macroglobulinemia: final analysis from the randomized phase III ASPEN study. J Clin Oncol. 2023:JCO2202830. doi: 10.1200/JCO.22.02830. Epub ahead of print. PMID: 37478390.
2. Tam CS, Giannopoulos K, Jurczak W, Simkovic M, Shadman A, Osterborg A, et al. SEQUOIA: results of a phase 3 randomized study of zanubrutinib versus bendamustine+ rituximab (BR) in patients with treatment-naïve (TN) chronic lymphocytic leukemia/small lymphocytic lymphoma (CLL/SLL). Blood. 2021; 138: 396.
3. Qin S, Chan SL, Gu S, Bai Y, Ren Z, Lin X, et al.; CARES-310 Study Group. Camrelizumab plus rivoceranib versus sorafenib as first-line therapy for unresectable hepatocellular carcinoma (CARES-310): a randomised, open-label, international phase 3 study. Lancet. 2023; 402: 1133-1146.
